# Fancy a Game of Football?: The Well-Being Benefits of Engaging Children and Young People in Out of Home Care in Sport

**DOI:** 10.1007/s40653-026-00910-6

**Published:** 2026-05-29

**Authors:** Gavin Breslin, Shirley Boyle, Mark Johnson, Stephen Shannon, Julie Patterson

**Affiliations:** 1 School of Psychology, Queen’s University Belfast, University Road, Belfast, BT9 5BN UK; 2 The Open University, 110 Victoria Street, Belfast, BT1 3GN UK; 3 Family Trauma Centre, 1 Wellington Park, Belfast, BT9 6DJ UK; 4 School of Sport, Faculty of Life and Health Sciences, Ulster University, Magee Campus, Northland Road, Derry, BT48 7JL UK; 5 Women, Children & Families Division, Northern Health and Social Care Trust, Bush House, 43 Bush Road, Antrim, BT41 2QB UK

**Keywords:** Trauma, Adverse childhood experiences, Exercise, Mental health, Adolescence, Soccer, Coping

## Abstract

Children and young people in Out of Home Care (OoHC) are amongst the most vulnerable and traumatised in our society. Meaningful engagement of these young people in services can be met with several barriers. To date, few studies have assessed whether programmes such as leisure time football can be effective in enhancing health and well-being, and offer an effective model for engagement of young people in mental health services. To assess the effects of a five-aside football programme (delivered in one Health & Social Care Trust in Northern Ireland) on health and well-being of young people and their carers. To offer ideas on how the programme could be developed to improve access to physical and social activities for children in care. A cross-sectional qualitative research design. Two focus groups were conducted immediately after a football session with an all-male sample, one with five male children (aged 12–22 years old) in OoHC, and the second with their male carers (*n* = 5). General Inductive Analysis (GIA) was used for thematic analysis and applied to focus group transcripts. A trauma-informed approach was applied in the design and conduct of the focus groups. Five main themes emerged from the focus group with young people, including: physical, psychological and social health benefits; motivation and enjoyment to participate each week; benefits beyond the football pitch; challenges of participation. Seven main themes emerged from the focus group with carers. Two themes were similar to those raised by the young people, namely: physical, psychological and social health benefits; and motivation to participate. Relational engagement was a primary outcome, for example carers viewed key barriers to participation, but felt that their professional relationship with the young person improved, as well as enhancing their relationship with other carers. The carers suggested improvements, but were also cautious of making any changes to the programme. Carers reported that safeguarding and risk assessment were perceived to be too stringent, but a necessary requirement. The programme was viewed positively in terms of engagement levels of young people, as was the transition of positive experiences “on the pitch” to (their) “off the pitch” experiences, alongside improved relationships and communication with young people. Football can improve relational engagement and effectiveness of service provision between carers and young people in OoHC, whilst offering health benefits. The provision of five aside football for children in OoHC is a low-cost, scalable initiative to complement existing therapeutic approaches.

## Introduction

Children in Out of Home Care (OoHC) are among the most vulnerable and traumatised young people in our community (Galvin et al., 2022). OoHC refers to the short or long-term care of children and young people who are unable to live with their families due to child protection orders or as a result of parents being unable to provide care or protection (Jones et al., 2011). In many cases OoHC is a last resort, and those children are more likely to have been neglected, suffered abuse, display more complex needs, have experienced a higher number of foster and residential placements, and have a higher number of critical incidents (i.e., death of a family member, and/or traumatic experience) (Galvin et al., 2022). Policy makers and practitioners globally are challenged to respond to the diverse and complex needs of children in care (Munford, 2022) as these children face multiple barriers to learning and social development, largely due to Adverse Childhood Experiences (ACEs) and attachment difficulties (Bailey et al., 2018; Galvin et al., 2022; Munford & Sander, 2019; van der Kolk, 2007). Furthermore, difficulties are compounded when service engagement is inconsistent or promised but not delivered adequately (Munford, 2022). For children in OoHC, behavioural, cognitive, social and mental health challenges are higher compared to the general population (Sariaslan et al., 2021; Sullivan & van Zyl, 2008). There is also extensive scientific evidence that ACEs increase risk for health problems in adulthood, such as depression, heart disease, cancer, obesity, asthma, arthritis, and substance misuse (i.e., smoking, problematic alcohol use, heavy alcohol use, illicit drug use, and cannabis use) (Desch et al., 2023; De Rubeis et al., 2023; Waehrer et al., 2020; Wooldridge et al., 2023; Zhu et al., 2023).

Furthermore, children in OoHC are at a higher risk of experiencing social isolation due to strained relationships with other family members, authority figures, and potentially being moved to communities where they know very few people. These young people may also have less developed social skills than peers, and see family or friends less frequently, which can impact on their sense of identity and belonging (Emond, 2014; Howard et al., 2023; McDowall, 2013; McMurray et al., 2011; Wood & Selwyn, 2015). They may also delay seeking health support when required, like seeking regular dental assistance, or seeing a doctor. This can be problematic as non-engagement/delayed engagement in health services can lead to minor health concerns resulting in longer term avoidable health problems (Bahanan & Ayoub, 2023). In many cases authority and regimented rule-based programmes or services may be off putting or viewed by the young person as unimportant, unattractive, oppressive, anxiety inducing or not beneficial to them. Young people who have experienced complex relational trauma may also struggle to engage with adults and peers in group settings that require a high degree of social interaction and communication skills (Hambrick et al., 2019). This view is supported in a recent literature review that found stark discrepancies between the ways in which interventions were framed/designed to support people affected by ACEs, and the types of support and services which young people described that they needed (Lester et al., 2020). This misalignment, or lack of listening to the views of young people regarding what their needs and preferences are, has affected sustained engagement and maintenance of support. It is apparent that more creative ways of engaging and then maintaining young people in trusted long-term services is required. This aligns with wider literature which highlights the need for service providers to pay closer attention to the voices of young people (Ungar et al., 2014). According to a review conducted by Wood and Selwyn (2015) young people want to create and maintain trusted relationships, want to be treated like their peers, be free to participate in their hobbies, participate in outside school activities, and avoid the stigma of being ‘cared for’. Similarly, the top three elements of a good placement according to children are: strong relationships; receiving social support; and participating in activities in their free time like sport and physical activity (McDowall, 2013).

Due to their prominence in social life and culture, creative ways of initially engaging young people can be achieved through sport and physical activity (Breslin & Leavey, 2024), and are viewed as an effective ‘hook’ to involve young people in mental health and well-being promotion (Swann et al., 2018). Team based sports have been shown to have positive benefits to mental health (Eime et al., 2013; Taliaferro et al., 2008). Those who had experienced ACEs and participated in team-based sports in adolescence showed less depressive symptoms in adulthood with improved social relations, physical health and self-esteem (Easterlin et al., 2019). In a study by Chandler et al. (2020) those with ACEs who took part in a strengths-based resilience intervention programme showed greater increments of positive change in emotional awareness than participants without ACEs. Such findings are promising, as sport and physical activity participation in youth may causally buffer against deleterious impacts of ACEs on health and well-being. In a recent systematic review of physical activity interventions with children and adolescents in OoHC, physical activity interventions were shown to address health concerns and according to the authors should be considered in policies and programme design (Wilson & Barnett, 2020). That said, ACE exposure in adolescence is associated with reduced odds of sport participation, so while sport is traditionally seen as an intervention for youth to build resilience, it may be appropriate to consider targeted interventions, especially for boys who experience ACEs (Noel-London et al., 2021). Additionally, these authors suggest that utilising a trauma informed framework within the world of youth sport may be of value when implementing programmes.

Within the Northern Health and Social Care Trust in Northern Ireland, a pilot project using a game of weekly 5-aside football (soccer) to engage young people who are in OoHC was conducted. To provide some context, in 2023, 22,875 children in Northern Ireland (with a population of approximately 1.9 million people) were known to Children’s Services as a ‘child in need (Article 17 of the Children (Northern Ireland Order 1995). 35,503 children were referred to Children’s Services, with over one- third being referred by the police (37%). During the same time period, 3,801 children and young people were deemed ‘looked after’ via Trust OoHC placements. This was the highest number recorded since the introduction of the Children (Northern Ireland) Order 1995: 22% of the children in care had been looked after for less than one year, with 32% looked after for five years or longer; just over four fifths of the children in care were in foster care placements (83%), while 7% were placed with parents, 6% in residential care and 4% in other placements (Department of Health, 2023). Focusing on children in OoHC, the 5-aside football programme was led by a Clinical Psychologist and Social Worker and designed to engage and maintain the interest of young people to attend, as well as improve the establishment of relationships between carers and young people. To do this, the young people play football with their carers and other professionals including Clinical Psychologist and Social Worker. The weekly programme has been running for 6 years.

The aims of the current study were to use a trauma informed approach to (1) assess the effects of the football programme on the health and well-being of young people and their carers, and (2) to identify how the programme could be improved for future delivery.

## Method

### Participants

Due to the small-scale nature of the study and challenges in recruiting young people in OoHC, convenience sampling was employed to recruit OoHC children and young people, and their carers. Carers are defined as residential childcare staff who act as keyworkers of the children/young people. A total of 10 participants were recruited to take part in two focus groups: One focus group with five children/young people attending football (aged between 12 and 22 years) and one focus group with five carers of children/young people who attended football with the young people (aged 24–44 years). The sample, and its size, were chosen as this was the consistent number who attended, and was exclusively comprised of males, (females had previously been invited to attend the football but had not opted to).

To recruit children and young people to the study, their parents were invited by the clinical psychologist (MJ) to provide opt-in informed consent. Contact with parents was initially made either in person or via telephone, followed up with provision of written information (with participant information and consent form), either in person or via post. The researchers’ contact details were provided so they could be approached to answer additional queries either in person, via email or telephone. Parents had two weeks to consider the request, and only if they provided written informed consent was their child/young person approached to take part. A stamped addressed postal envelope was provided to facilitate return of the consent form. In the event that a parent was deceased or estranged from the child/young person, consent was obtained from the statutory social worker, wherein the Health and Social Care Trust is the corporate parent and has legal parental responsibility under the Children (NI) Order (1995). Children/young people and carers were approached by members of the research programme team to participate (JP, MJ). They were provided with written information about the study using a Participant information sheet, and had points clarified and questions addressed to ensure understanding of the content. Prior to completing the consent process, the researchers checked to ensure that participants were happy with the aims of the study, their obligations and that they understood the researchers’ responsibilities to them. It was made explicit that consent can be withdrawn at any stage. A similar recruitment approach was taken with the young peoples’ carers.

### Design

**Fig. 1:**
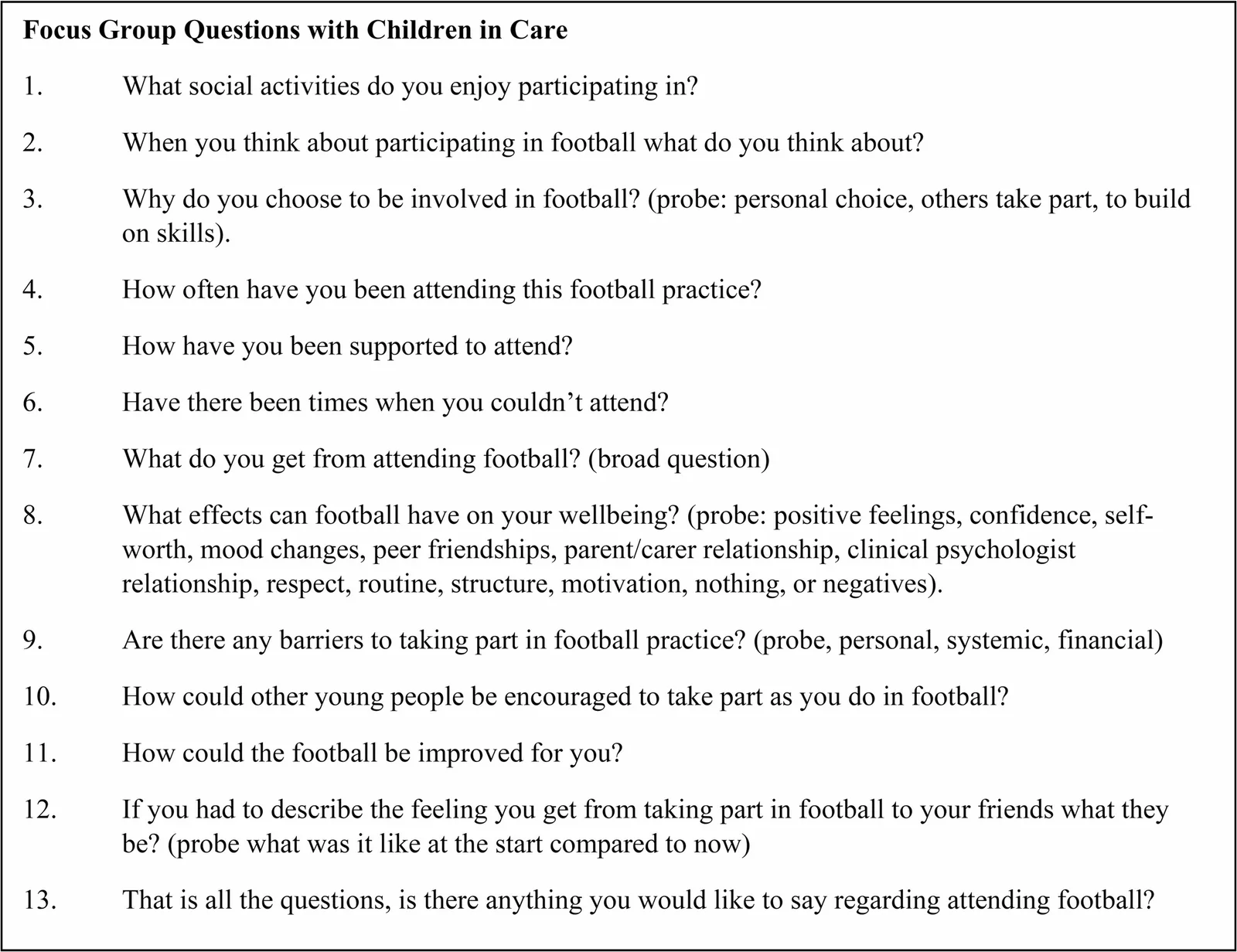
Focus group questions with children in care

**Fig. 2:**
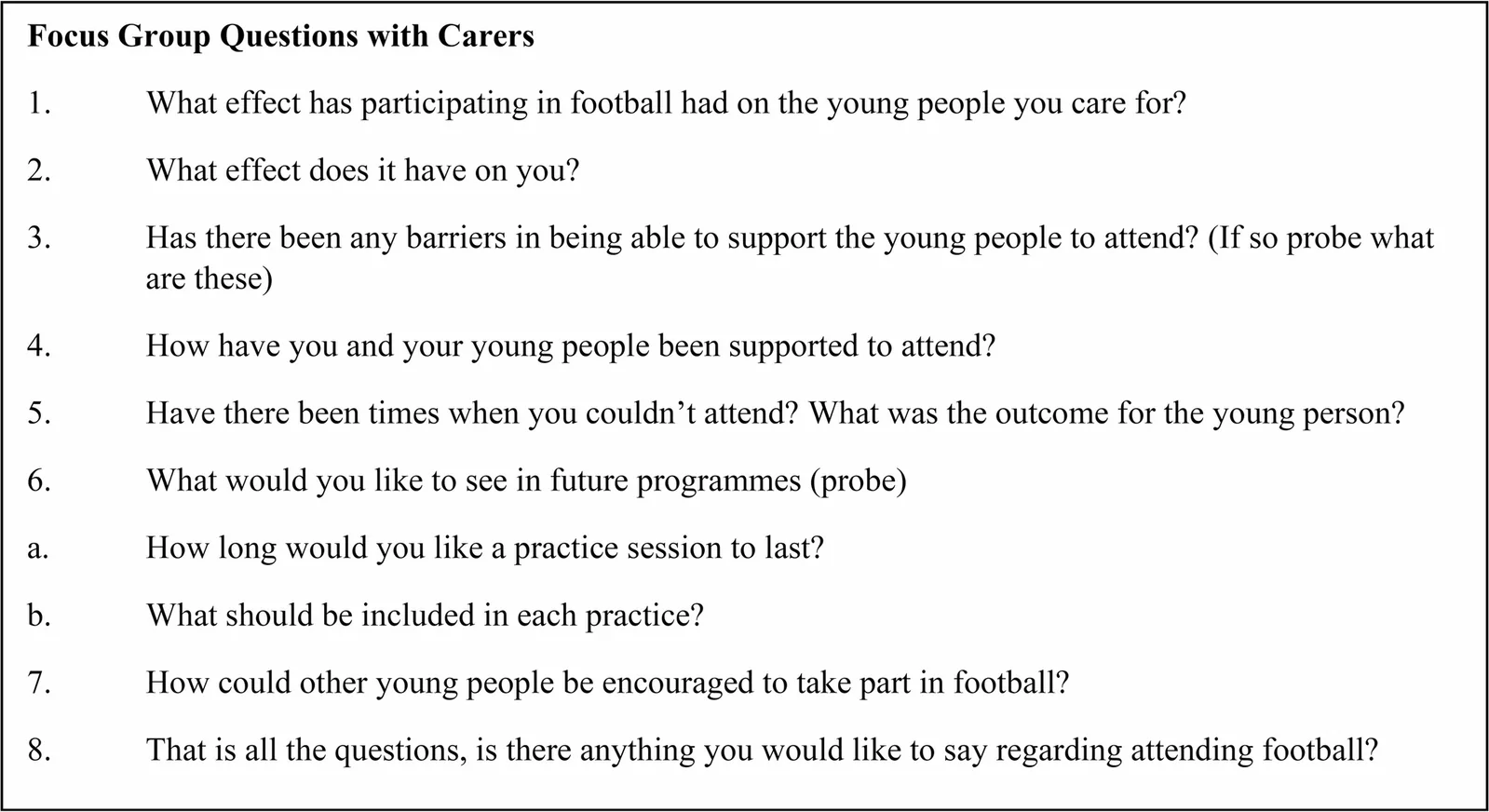
Focus group questions with carers

A retrospective cross-sectional qualitative research design was employed wherein two separate focus groups were conducted, one with children in care, and one with carers. The focus group for the young people in care were guided by a semi-structured schedule of questions as outlined in Fig. 1. Questions for the carers are available in Fig. 2. These were developed by the research team through an iterative process, ensuring alignment with the research objectives and ethical requirements. The focus groups were facilitated by GB and SS. The benefit of the semi-structured questions was that open-ended questions afforded participants the opportunity to express and expand their thoughts more fully (Thomas et al., 2005).

Both focus groups lasted 38 min in length. With participant consent, focus groups were audio recorded using a Dictaphone (Philips, VoiceTracer Model 660) and transcribed verbatim by SB, and then checked for accuracy by a second researcher (GB).

### Five-Aside Football Programme

The five-aside indoor football programme lasted one hour and took place every Wednesday evening in a local leisure centre. The five-aside team of carers, psychologists, social workers and young people were mixed each week. The young people were transported to and from the leisure centre by residential care staff or foster carers. The games were added to the schedule for the young person’s weekly activities and was prioritised, when possible, in homes were staff was impacted by the availability of ‘footballers’ on the rota, or the needs of other young people in the home. Not all staff in residential care play football, and while the home endeavoured to put carers who participated in the football on the rota for a Wednesday evening this was not always possible. However sometimes staff came in to work on their days off to accompany young people to the football games. Carers were responsible for letting the organiser know if they or the young person would be attending that week. The costs of hiring the sports centre for the game and travel were funded by the Northern Health and Social Care Trust.

### Data Analysis

A General Inductive Analysis (GIA) approach was adopted to interpret focus group data. GIA is an approach that aims to derive more general concepts through the interpretation of raw textual data (Thomas, 2006). It involved identifying patterns, themes, and categories from the data without starting with a predefined theory or hypothesis. This approach was deemed appropriate as it: (a) enabled GB and SB to condense raw textual data into a summary format; (b) facilitated the creation of linkages between research aims and summary findings from the raw data; and (c) was used to inform a framework for interpretation of participant views. Furthermore, inductive analysis was considered appropriate as there is little available information and theoretical models developed on this population where we could deductively fit our data.

Trustworthiness of the data, analysis and final themes were established through a number of checks conducted throughout the study as advised by Sparkes (1998). The data was transcribed by SB. Both GB and SB read the transcripts, agreeing an overarching thematic framework for coding (related to the research questions), and noted emerging themes to be developed into codes. They swapped their coding notes and met on three occasions to review and refine these into one agreed set of codes. The study included extensive participant quotes to enhance the validity of the findings, and illustrate the codes that had emerged from the data. All raw-data quotes were subjected to an audit trail that included (a mapping from the participant’s spoken word to theme creation); and finally, detailed discussions were held between GB, a registered psychologist, and SB, a registered social worker, in order to explain and challenge emergent themes.

Although the focus group lasted 38 min, saturation was achieved due to the focused nature of the questions, the small number and relative homogeneity of the participants, and the structured facilitation of the focus group. Saturation does not depend on the duration of the session but when no new themes emerge (Guest et al., 2006).

### Ethical Approval

Ethical approval was provided by the Office for Research Ethics Committees Northern Ireland. Approval number 21/NI/0141. Given the study involved children, particular care was taken to ensure trauma-informed considerations were part of the design. Participant information and distress protocol was developed as part of acquiring ethical approval. Furthermore, one of the research team members (MJ) is a clinical psychologist, who was well known to the young people and carers via his therapeutic support and consultation role in the Trust. Ethical considerations about young peoples’ trauma histories meant they could advise about the suitability of inviting young people to participate.

## Results

### Responses from Children and Young People in Care

The thematic structure was aligned to the research questions examining the experiences, benefits and challenges for young people attending football sessions. Five main themes emerged from the focus group with young people. These were: health benefits, motivation to participate, benefits beyond the football pitch, challenges of participation, and improvements. Sub themes are described below, and a summary of themes are presented in Table 1.

**Table 1:** Themes from children and young people

Health Benefits	Motivation to participate	Benefits beyond the football pitch	Challenges of participation	Improvements
Physical health benefits	Initial Uptake: Why Play football?	Communicate better	Physical effects	Develop a team- versus keep as is
Psychological health benefits	Adherence: Feeling supported to attend football	Confidence	Acting silly (e.g., disrupting session, making fun of each other)	Outside more
Social benefits		Friendships with staff		Longer session

### Health Benefits

Physical health benefits of participating in the football programme included improvement in perceived muscular strength and physical fitness. Participation was seen to:

‘burn up energy’ and ‘helps you build up your stamina’.

Psychological health benefits included raised mood:

‘burn up energy’ and ‘helps you build up your stamina’,

controlling temper:

‘It’s definitely helped me controlling my temper.’; improved confidence, ‘boosts your confidence like’,

concentration:

‘You can concentrate on one thing. ‘take your mind off things going on in your head’,

and stress reduction:

‘Just releases stress’.

It was also recognised that participation in football can reduce aggression:

‘it gets all your aggression????out’ and ‘improves sleep’.

The **s**ocial benefits included making new friendships as well as:

‘Getting on with everyone else’,

‘Getting on with everyone else’,

‘You just get to socialise and talk to people’,

and

Building relationships.’

### Motivation to Participate

The participants were motivated to take part in the football programme along with their carers, social workers and the clinical psychologist. Initial reasons for uptake of the programme and adherence were discussed. Reported attendance duration by participants ranged from two months to five years.

#### Initial Uptake: Why Play Football?

Young people commented on why they started football:

‘I’ve always enjoyed football and I think coming up through school and stuff, not being the fittest person, you were always last picked, you weren’t picked in the team, you were kind of left behind. Whereas you’re coming up here and everyone’s getting to participate, everyone’s getting to have a go. There’s nobody excluded from it.’

One participant who initially got involved because they like football has been participating each week for the past five years. The motivation to play also appeared to be related to the attraction to this particular football programme, for example one participant who had played at a club previously and dropped out said:

‘But here, everyone’s here to have a bit of fun, kick about. There are no expectations of anybody. You’re just there for the purpose of playing football and having fun at it. So, there’s sort of a, less of an expectation for you to do well.’

Similarly, another young person said:

‘At the beginning I was a spectator. I’m not a very outgoing person. I’m not really much for socialising myself. I’ll admit to that. But whenever I came and I seen how everybody else was, and how it was, yeah, I wanted to come back again.’

Another young person said they got involved because of how they were recruited:

 ‘I was recruited through a psychologist- and word of mouth.’

#### Adherence: Feeling Supported to Attend Football

When asked about adherence to the football programme all said that they felt supported to be there and by keeping coming it ‘keeps you out of trouble’. It was also felt that by maintaining attendance there are many social benefits as already reported.

### Benefits Beyond the Football Pitch

Benefits beyond the pitch included better communication skills, improved confidence in other social contexts, and development of friendships with staff.

#### Communicate Better

The young people reported feeling more able to hold conversations with other people:

‘I think it helped me a bit … thinking back at it now, if I hadn’t gone to the football and learned to communicate better with people, because whenever I was at college I … I guess coming to the football and stuff you always talked to everybody, sort of helps you with your communication skills. And then going to uni, you’re meeting new people, completely. It’s like I now feel more confident when I talk to new people. Getting to know new people and stuff.’

It was highlighted that conversations during football were fun and engaging:

‘I think if I see XX through the week, we have a wee bit of banter about it (referring to the game). You know, maybe winding each other up … It’s definitely talked about through the week, and as XX mentioned there, I think the fact that we have been going every week on a Wednesday is becoming a routine nearly.’

The sense of camaraderie that resulted from better communication and enjoyment of being with others who attended football was also evident:

‘Yeah, back whenever I started … there was a three of us in the car that came up every week. We were all in the same area at the time. So we all got along. Got friendly and stuff really well. If I met them in the street today, I’d probably still say hello.’

#### Confidence

An example was given of how learning skills of socialising during football improved their confidence in other contexts, and in one case, supported the young person to support others:

‘There was just something about it, so coming through college and stuff like that, I was very closed in to everybody … I wouldn’t talk very much about how I was, or what I was doing and stuff like that. But going up through it [football], getting to know people that were there, and then new people coming in, at that point it felt easier to talk to new people coming in. You go up and you try and make them more comfortable … so I would just go out there, start a conversation, make everybody feel comfortable … cause then everyone’s going to get along easier.’

Similarly, it enhanced their ability to empathise with others who are new to groups and social situations, by drawing on their own experience and development:

 ‘Yeah, you’re going to adapt those sorts of skills that you need to learn … people coming in, they probably going to come in, maybe they’re a bit shy, they’re not confident talking to new people. So having done that yourself, when you do go into a group you can kind of adapt that skill you’ve learned, to try and make other people feel comfortable, in different situations.’

#### Friendships with Staff

The relationship with the psychologists, social workers and carers was shown to improve, and outside of the football context this was evident:

‘It’s good aye. I always had a relationship with everyone here like. When referring to football and the session it was felt that ‘Aye it’s talked about during the week’, aye.’

### Challenges of Participation

There were some less positive impacts of taking part. The physical side effects of high intensity exercise left some of the participants:

 ‘Feeling absolutely exhausted after it!’

It was also apparent that on occasions an issue could arise when some young people started:

 ‘Acting silly.’

‘just waste people’s time. Just starting a fight over silly things, or just wasting time, and just wasting half an hour of football sometimes, or 10 mins. We could be playing football, there’s boys just arsing around, or acting the lig.’

For some participants this detracted from the experience off the match.

### Improvements

When asked what could be improved about the programme there were several suggestions that included:

‘If we were outside more’, ‘Longer session’ or playing on ‘Real grass pitch’ and to increase the competitiveness and play other teams, to ‘Develop a team’. Overall, when asked about what changes would be welcome, there were mixed views and most participants felt that they should ‘Keep as is’. This was because they felt they had been a part of something that provided a safe place that was enjoyable for them, and if any changes were made it could lose its attraction and purpose. This suggests they felt a sense of belonging and ownership of the group.

### Responses from Carers

Seven main themes emerged from the focus group with carers. These include health benefits, motivation to participate, barriers to participation, professional relationship with the young person, benefits to staff, improvements versus no change, and safeguarding and risk assessment. Some themes were similar to those raised by the young people in care. See Table 2 for a summary of themes.

**Table 2:** Themes from the carers

Health benefits	Motivation to participate	Barriers to participation	Professional relationship with young person	Benefits to staff	Improvements versus do not change	Safeguarding and risk assessment
Physical health benefits	Why play football?	Football Skill Level	Relationship development	Meet staff and Kids from other care homes	Venue	Risk Aversion
Psychological health benefits	Providing support to initially attend	Staff availability	Conversation starter	Provides Consistency	Skill development, training and circuits	
Social benefits	Effort to ensure it happens: Adherence	Other competing activities	Change of Setting- it’s informal		Tournaments	
	Consistent venue	Role of staff- as both a barrier and enabler			Broaden to other type of activities	

### Health Benefits

The physical health (e.g., cardiometabolic) and psychological (e.g., stress reduction) benefits of the programme were recognised by the carers. The carers described more health benefits for the young people than the young people directly identified:

‘I think it’s gives them a bit of an outlet. Throughout the week to get out and playing football. I know myself, playing sport and stuff, you just, maybe pent up, sort of, not saying anger, aggression, but any sort of pent-up feelings, just get them out when you’re on the pitch and you leave it there.’

The football programme was also perceived by the carers to build self-worth and keep bringing the same participants back:

‘Boosts confidence. Like a lot of these guys would have really low self-worth and it, I think, they get to understand that people care about them, and bring them along. There’s been people coming for a long time. This has been going for a long time and I keep seeing the same faces coming back.’

It was also highlighted that:

‘A lot of kids in care suffer from different things, like drugs or alcohol. This can give a much more natural high than any of that can give.’

Similarly, it was felt the programme helped the young people to emotionally self-regulate:

‘And a lot of them really struggle to emotionally regulate. They find it tough at times to emotionally regulate and this definitely enables them to, as X says, develop the life skills, but also to develop some level of emotional regulation. It takes a lot for our young people to prevent them from kicking off at for the smallest wee thing. But there’s something about sport and exercise that allows them that level of restraint that they maybe don’t have in other parts of their lives.’

There was much discussion about the social benefits of football:

‘Socially definitely. I think they get a bit of camaraderie from it. They learn a lot of life skills from it because they have to share. They have to take turns. They have to work as part of a team. And it’s true that a healthy body makes a healthy mind.’

The informal setting of playing football was viewed to be supportive for developing social skills:

‘I think the young people that come are a marginalised group of young people who have experienced a whole range of adversities and challenges and I think this opportunity gives them a wee sense of being, sort of just flitting into society. Being normal. Not having those taboos or stigmas attached to them that sometimes they may face in other settings.’

This was further supported by views that the young people showed more politeness or gentleness in their manner in playing:

‘There’s a politeness about them as well. Like they call everybody by their Christian name, everybody shakes hands afterwards. It’s all very mannerly, gentlemanly.’ (group laughter).’

It was acknowledged that football provided young people with experiences of social group norms and behaviours:

‘… our young people they live in groups. They often live with other young people but you find that sometimes that’s really tough, whereas this is a group that’s kind of, it’s natural, it’s organic in some respects. You know people do come voluntarily. And that allows them to kind of understand those dynamics that most groups sort of face. The goods and the bads and the indifference.’

This was further supported by the view that football provides a sense of normalisation, that is important to identity formation of young people in care:

‘I think, ye know they’re everyday in such an artificial environment. Especially in residential. You know this is much more natural … brings a bit more normality in their life which is what every child should experience.’

‘It’s quite formal in residential. You know, have those 1–2−1s … playing football, you know, just more like teammates. And that power imbalances goes way down as well.’

It was also suggested that the football matches enhanced social cohesion and inclusion:

‘It creates a connectedness and I suppose a sense of belonging for those guys. That they belong to this group that we have.’

‘No matter where you come from, shape and size, ethnic background sort of thing. Like it’s for everyone, not just one specific person. Everyone can enjoy it.’

An example of how the programme was used to bring siblings who had been separated together again was reported:

‘…so I used the football to get the three of them together, and it worked. There was no animosity at all. They just played away together… one’s still coming here.’

### Motivation to Participate

#### Why Play Football?

The carers felt that participants were motivated to play football because it had many short-term gains, it was informal, it was fun and was preferred over other types of exercise:

‘I think they enjoy the football because there’s short term gains because you win, and you score goals. Contrast to that is working out and doing circuits, it’s more long-term gains from it.’

‘The young people like the element of fun, and they like the element of the quick gain, being able to do it… It needs to be something that they can actually manage. Because a lot of them like to go to the gym … and it’s the novelty … it’s not like they’re going in and doing a proper session, but they like to try it out … but it has to be in some way doable for them.’

#### Providing Support to Attend

The carers put a lot of emphasis on how they support young people to maintain their motivation to attend football each week. They recognised that attendance is voluntary, but that once it is scheduled in the calendar they do what they can to ensure the young people are transported to the venue and are free to attend (usually every Wednesday evening):

‘Yeah they come voluntarily. There’s nobody forcing them to take part although we would encourage it a lot.’

‘I would say plenty of prompts, plenty of encouragement and staff providing the lifts. Staff transport them from the homes.’

‘But I think people go all out to try. If you have somebody who’s good at something, we do our best to promote that.’

The support from others (such as foster parents, other professionals and organisations) is important to maintain the momentum and motivation to ensure sustainability of the football sessions each week:

‘… I think the REC team as well plays an important role in there as well because they kind of somehow do the linkage. Because … they would be reminding the kids as well doing that and saying look you know footballs on a Wednesday night. It gives the kids kind of a focus on, you know for their particular week. You know they know.’

The persistence in encouraging new members is also important:

‘… one of the other young people in the home, I says ‘come on down to football tonight and get yourself out’. Like he hasn’t came any other nights, and he has been honest, but I didn’t expect him to come. But he came. You know, so it is just that persistence and always providing the opportunity… to everyone.’

It was also noted that staff often gave their free time to participate and promote engagement with football, sometimes coming into work on their days off. This was particularly evident for tournaments and when matches were organised between different trusts.

‘Yeah, the majority of young people like live in residential, so it takes a commitment from the teams to actually plan shifts around transporting young people down and ensuring there’s enough staff available. So there’s a lot of effort that goes into actually trying to prioritise attendance, where it can be facilitated. Which demonstrates … the importance it has for staff within homes. Staff do see the benefits that it has for the young people.’

### Barriers to Participation

#### Football Skill Level Was Viewed as a Barrier to Taking Part

‘I have brought a couple who weren’t really footballers, and I don’t think they realised how seriously we all take it. And it wasn’t for them. They came a few times, and they realised it was just too quick for them.’

‘I think sometimes it’s a confidence issue or a self-esteem issue, or it’s a bit of a fear of the unknown for a lot of the young people.’ …And then what happens is they usually volunteer to go into nets.’

#### Staff Availability

A further barrier is staff availability to attend the programme:

‘Lack of staffing has probably been a bit of a barrier. Having people available to come up.’

It was also mentioned that some staff are more interested in football, so would be more motivated to ensure the young people attended.

#### Other Competing Activities

There were other activities that negated young people from attending at times:

‘I’d a wee boy today and he said he was going, he promised a couple of ‘Reach & Connect’ guys he was going. He was going all day long, until somebody mentioned ice skating … And he chose ice skating.’

A restriction was that some young people are more interested in activities other than football, or like their own individual time that the carers were cognisant of:

‘Some of the young people just like to stay in their room and gaming too. It’s just the way it is I suppose.’

#### Role of Staff- as Both a Barrier & Enabler

While it has already been highlighted that staff availability and the context of the care environment can impact on facilitating attendance at football, relationships between staff and young people is also a factor:

‘Some young people will go with any staff member, and that’s just because they’re able to. Some young people will have their staff members that they’re close with, they’ve got a good relationship with, they feel safe with, and they’ll not go unless that staff members there. So it’s very unpredictable. I suppose, dependent on the young people I think, would depend on whether they would come down themselves or whether they would rely on a particular staff member.’

The unpredictable nature was also evident in a carer indicating that consistency in staff being available and showing enthusiasm was important in keep routine of attending football:

‘… from a staffing point of view … you might get Joe Bloggs [referring to a young person] that goes into a particular children’s home, and because of a range of different barriers isn’t able to engage for a number of times. But actually, then they kind of have a bit of enthusiasm, but because they haven’t engaged before, because they haven’t shown interest, there might be that kind of complacency that sets in from a staffing point of view as well.’

This participant asserted that a young person who is initially reluctant to go to football, may become interested, but this can be overlooked by staff, because the young person originally presented as disinterested.

### Professional Relationship with the Young Person

Developing personal and professional relationships with young people was identified as a significant outcome of the programme. It was reported that football assisted with the development of relationships, was a good conversation starter and provided an atmosphere/climate that was less formal for young people to engage with their carers and the psychologist. All of the carers reported that the programme was beneficial for developing relationships with the young people:

‘Good for your relationship. It’s great for you’. Similarly, there’s something about the 1–2−1’s though too, isn’t there, like for yourself and XX. For yourself and XX? Like that’s kinda been important.’

#### Conversation Starter

The programme was seen as a way of engaging young people and their carers in conversations:

‘Big time. Yeah. Cause I would see XX today and on a Saturday. And on Saturdays we’ll talk about football. Football’s always a topic of conversation between us, so. When I go to pick him up, ye know for football, every time he asks, so it’s just that routine, ye know.’

The programme was also seen as a way to develop a bond:

‘I definitely think there’s definitely a bond…just if you were to see somebody in a work capacity, or out in the street, you’ve done something together, it’s an easy conversation starter. You know, you can share or reflect back on a memory or a bit of craic*. It’s easier to start a conversation when you’ve done something together with somebody. It’s a positive.’

#### Change of Setting- It’s Informal

The informality of the football was seen as a benefit in engaging with those in care:

‘Young people get to see us maybe in a slightly different light. Get to see a different side of us. Ye know, can have a bit more fun with us maybe, than if we were working.’

Carers also recognised that the range of roles of the carers was varied but everyone was treated the same:

‘Even the range of roles everybody’s had that comes here is great. You know, it doesn’t matter what your job role is, you’re welcome here and everyone’s treated fairly.’

‘It definitely builds relationships. The most important thing working with young people who live in residential care or who have experience of care, is relationships … This is a natural environment. The power imbalance reduces. It’s not like XX is a worker … it’s XX my team mate on the team … there’s something just so natural about that that allows the natural progression of the relationship that strengthens how you work with the young person beyond this environment.’

Similarly, young people benefit from seeing beyond the professional to discover the person:

‘Young people like to see you not with your formal hat on. They like to see that your human. They like to see your quirks … Like you’re someone that they can relate to. It’s not … like a bit stuffy in your work attire. There’s just something about these environments that allow young people to engage with you a wee bit differently.’

‘And it takes them out of that working environment. They don’t see it as the worker or you’re just teammate and you’re playing with them.’

‘It’s the informality. Its more relaxed because there’s an activity and you’re taking part in an activity, a physical activity. So it’s not like a formal 1–2−1 engagement.’

### Benefits to Staff

Several benefits were highlight from staff participation, these included meeting staff and young people from other residential homes, and providing consistency in care.

#### Meet Staff and Young People from Other Residential Homes

Extended social encounters provided through football were highlighted:

‘I think one of the things that I’ve really enjoyed is getting to meet the other staff from the other homes as well. Because it helps to build those relationships too. You know what I mean. Because we all sort of do in a way intermingle and come across the kids at different points in time.’

‘Something I get out of it I, at meetings you hear all the names of the kids, but now I’ve met a few of them, and you see them in a different light.’

‘We get as much out of it as the children do. it goes back to the thing about relationships. It’s a really natural way to build relationships with young people.’

While the research did not set out to explore benefits to staff, it was refreshing to hear about how staff enjoyed it as well.

#### Providing Consistency

Staff commitment and their appreciation of the benefits football brough to the young people served to reinforce the need for consistency and sustainability:

‘This is one constant. We are going to make this happen on a regular basis. I think it’s a great way to build relationships, from young people to adults, everybody’s. Like it’s inclusive, you know, people of all ages, all abilities. They’re here to play. There’s no fear of judgement. You’re in a safe space. So, I think it’s probably just that constant. You know, recurring thing. I think that’s the best part about it.’

Providing consistency could be challenging when there was occasions of low uptake amongst the young people, but staff persisted:

‘There’s been periods when numbers have been really low, maybe like 3 aside. But we never cancel it. here. Covid was probably the worst thing that happened, because we were flying before COVID and it’s only sort of just getting back into the swing of it now.’

‘We all know the night and the time, and it doesn’t change. The venue is normally here and that’s been the same for years.’

‘The hall wasn’t open for a couple of weeks and actually the football didn’t proceed because there’s a relationship with the centre and they know. They’re familiar with the staff and the young people coming.’

### Improvements Versus ‘No Change’

There were several suggestions to change the programme to enhance it, however, similar to the young people, carers were aware that any alterations may hinder or adjust what is currently working. It was felt that what has been created for the young people in their care is given detailed consideration before making changes as the ‘enhancements’ may detract from what is currently effective. These are some suggestions for improvement.

#### Duration/Schedule

Carers shared views that the duration and frequency worked for the young people:

‘I think an hour hits the spot generally. Cause there’s nothing worse when you’re unfit and you’re playing more than an hour.’

‘I think once a week works.’

#### Venue

The venue was considered to be appropriate:

‘This was a good venue, cause it’s central to all the homes. There’s not a lot of travelling involved for everybody,’

and important in contributing to stability for the young people:

‘The children anyway get comfortable coming to a certain place. They know it, they’re familiar with it. And so, taking them somewhere else then they’re out of their comfort zone for a wee while and it might just put them off.’

#### Skills Development

There were mixed views of orientating the programme more towards developing fitness and football skills:

‘Like I don’t know what the appetite would be like, but it would be that skills development, and working towards some of those opportunities which generally go down brilliantly.’

There was a suggestion to extend and bring in circuits training to enhance fitness:

‘Thinking about a circuits evening. A guy from the XX team could deliver.’

However, there was a counter response to suggestion of fitness circuits:

‘Not sure if you’d get the same… numbers (others concur).’

‘I think the kids would struggle with it … and wouldn’t get the same feeling that you’d get from being part of the team. Part of a group, but I think it adds another element of formality to it and I think that’s why our kids struggle.’

Avoidance for change and an eagerness to protect what has been generated with the young people was shown in this quote:

‘I think the game works well. I suppose if you start bringing in cones and dribble and stuff, It’ll breakdown for them the interest.’

#### Tournaments

There was some discussion about past football tournaments with scope for developing this again:

‘I would say, some scope there for working something out between all the different trusts together. Bringing something together for a bigger tournament type exercise.’

#### Other Types of Activities

Several other sports were suggested, but these also came with child-centred considerations:

‘I think you need to make it as informal as possible …we’re always looking for our kids to get involved in youth clubs, or something in their local area … maybe a drop-in on a particular night, ye know, where the kids could come in and do something informal. We’re talking about table tennis or something like that there. Just an area or space that the kids can all go… and get a bit of pizza, chill out … A bit like a youth club but informal for them. You need to start somewhere like that … to try and get the young people in, to feel safe, and then for those kids that aren’t really willing to come, into a big massive leisure centre and meet totally everybody else.’

‘A 1–2−1 coaching session, or 1–2−1 taster session of come down and we’ll get a chat with them, and we’ll show them what the facilities is like, what the sport is like, so if you done that with football with somebody who wasn’t overly confident, or wasn’t sure about their ability, it may encourage them.’

### Safeguarding & Risk Assessment

The carers gave a lot of consideration to how they have to manage risk and ensure safeguarding of the children. There was a view that perhaps on some occasions if there was a risk they would avoid the activity, but on some occasions felt they may be over assessing risks:

‘In our world and it is going back to the kind of formality, but sometimes we do risk assess a lot. Based on who’s going on Wednesday night. And is that the right young people to promote to go as well because there’s maybe concerns around them linking up. They’ve maybe been vulnerable to exploitation, where there’s maybe risks around aggression. So you would find that those are barriers … as a professional group … we are sometimes hesitant in terms of who do you agree should go down and who shouldn’t, based on who we know has potential risk.’

Some concerns were given to who should and should not be brought together for football:

‘Yeah, two young people coming together and knocking ten bells out of each other really. Or going off and getting drink and drugs, or running away.’

‘I can only recall once when it became physical, but one wasn’t a regular attender and used the opportunity … to come up to have a go at someone else … A couple of weeks ago they used the opportunity to come along here then because it was handy for XX train station) to disappear! And took a couple of the other footballers with them, so that was something we had to take charge of and control.’

This example demonstrates the challenges staff encounter when things go wrong, and treading the balance between care and control. Despite these isolated examples, staff work to prevent difficulties arising, while enabling the continuation of the programme that provides a good experience for the young people.

## Discussion

The aim of the present study was to explore the health and well-being effects of taking part in a weekly five aside football programme among children and young people in OoCHs, and explore avenues for potential improvement and scalability of the programme. Overall, findings from two focus group interviews among young people and their carers revealed a consistently reported, positive, benefit of the weekly 5-aside football programme in improving young people’s physical and mental health. The study also highlighted how it garnered both initial uptake of, and sustained motivation that complemented young people’s engagement with services and enhanced relationships with their carers. These findings extend research on the contribution of sport to mental health, wherein many can forge social connections, realise a sense of purpose, and develop psychologically through participation (Breslin et al., 2019; 2024; Swann et al., 2018). Additional coherent themes among young people and their carers noted how the simplicity of 5-aside football, and its enduring presence in their lives, has offered them a safe and sustainable social and physical activity programme that requires minimal change – a goal that has historically been very difficult to achieve via standard healthcare practices among children and young people in OoCHs (Galvin et al., 2022).

Proposed principles of trauma-informed care emphasise the importance of safe, consistent, and trustworthy relationships to counteract the negative developmental impact of traumatic experiences (Bunting et al., 2019; National Child Traumatic Stress Network, 2016). Themes arising in the current study suggest that predictable and routine opportunities to connect with peers and adults within an informal setting via weekly football enabled a greater sense of inclusion, belonging, and confidence for some young people. Also arising in the discourse were examples of young people and carers reflecting that coming together in routine shared physical activity enabled them to better regulate emotions and communicate effectively with others; developmental competencies often observed to be negatively impacted via childhood trauma experiences (van der Kolk, 2007; Perry & Dobson, 2013). Benefits of social and relational connection between adults was also discussed by the carers, which although not intended as a core focus of this study, may allude to protective elements of the caregiving role that could serve to counteract some of the potential effects of vicarious traumatisation (Middleton & Potter, 2015; Molnar et al., 2017).

Some of the present study’s subthemes highlighted important caveats to the maintenance of successful engagement, and unintended consequences of participation, which will be discussed herein. In a review of the health behavioural sciences literature, Michie et al. (2014) reported that successful sustained engagement in interventions remains elusive. In Michie et al.’s (2014) COM-B model, successful engagement is theorised to be a function of an individual (i) perceiving themselves as both psychologically and physically capable of doing so (i.e., capability); (ii) believe they have the social and physical opportunity for the behaviour, (i.e., opportunity) and; (iii) feel the desire or recognise the need to perform the behaviour over other competing behaviours (i.e., motivation). While COM-B was not used in the design of this programme, exploring the effects and adherence to the 5-aside football programme using these COM-B components offers some insight. Specifically, the health benefits of participation in 5-aside football were widely espoused from both the young people and their carers, and ranged from improvements in emotional regulation (e.g., aggression), to enhancements in mood and physical strength (however carers articulated these changes with more finality than the young people did). While these findings are consistent with a corpus of literature on the positive role of physical activity for health (Biddle et al., 2021), significant emphasis was placed on the relaxed nature of taking part in the activity and therefore limited emphasis on competition and skill, but rather on enjoyment and socialising. These findings are consistent with an integrated conceptual model for young people (Lubans et al., 2016) that specified a range of potential direct and indirect links between sport and physical activity on mental health, some of which include how sport and physical activity provides opportunities for social interaction, enhances self-efficacy, and the supportive social context in which physical activity is practiced. These features were captured by a participant who highlighted that: “*everyone’s here to have a bit of fun*,* kick about. There are no expectations of anybody*”. Such lowering of expectations has also been argued as a crucial component in minimising shame and enhancing relational connections with traumatised young people who often struggle with the implicit rules and demands of social interaction whilst experiencing the effects of stigmatisation and criticism of others (Holden et al., 2010; Izzo et al., 2016).

Given the reported successful adherence to the programme, key characteristics of the programme related to the psychologists, social workers and carers, and participants themselves, ensuring inclusion and participation were likely essential for both (a) initial uptake and (b) sustained motivation for the initiative. Notwithstanding the reported inclusive nature and relative simplicity of the programme appropriate for scalability, some carers reported that other children may have lacked basic levels of physical fitness and football skills, which may have affected their confidence to participate. For such reasons, football as an activity itself, and sports generally, are therefore not preferred among all young people, wherein lifestyle activities (e.g., walking) or recreational fitness classes (e.g., gym, running, aerobics) could be considered complementary physical activity provision (Ihle et al., 2021). Some carers reported that such activities would however be best placed as in addition to football, as they would be unlikely to garner the same motivation from young people.

Furthermore, much of the success of the programme related to the reported provision of opportunity through transport, regular bookings of sessions, and suitable/appropriate venue and time. Linking back to Michie et al.’s (2014) COM-B model, the necessary social and physical opportunities are essential for ensuring sustained motivation for behaviours. From an organisational standpoint, having the support of senior management, psychologists, youth workers and carers in the delivery and financing of the programme was essential.

## Conclusion

This study provides an insight into the experiences of young people and their carers participating in a football programme, the impact on the health and well-being of young people, and the (unexpected) benefits to the carers as well. The potential to expand and develop this programme were explored, whilst recognising the importance of consistency and continuity in retaining features of the existing design. The results of this study, pay attention to an often-neglected aspect of children’s needs in care in social research. The findings have the potential to inform improvements in service delivery to this population, considering their generic fundamental psychological needs to socialise, have fun and play within a trauma-informed and risk-managed context. The study also highlights considerations for designing social activities for children in care, and strengthens the need to listen to the voice of young people in programme design. It may also provide useful suggestions for service providers considering the development of not only a football programme, but other types of sports and social activities.

It should be noted that the data collection for this study was conducted in the wider context of the COVID-19 pandemic, and after extended periods of lockdown. Therefore, the availability and uptake of the football programme experienced some disruption and by extension, recruitment and participation in the study may also have been impacted by this. A limitation of the study was the all-male sample. The findings reported in this paper were part of a wider study that was originally designed to include football and another social activity; a singing group (choir) for looked after children. However, the choir element of the programme was affected to such a degree by the pandemic, mainly due to the government restrictions that it was not reinstated after lockdown, and was therefore unable to be part of this project. As a result it would have been useful to consider alternative activities for children in care (beyond football with its somewhat inherent gendered orientation to favour males). This could have reflected the wider interests of young people in care (such that the singing group would have catered to those interested in music), which was the original aim of this project, to examine two distinct types of social activities and their impact on young people.

The focus groups comprised a small number of respondents from one health and social care trust area in Northern Ireland, therefore the authors are not claiming the study is generalisable. However, the findings from this study merit attention, given the lack of research in this field, to understanding the experiences of looked after young people and carers. The findings highlight some important themes in considering holistic needs of children in care, and the need for ongoing exploration, development, and potential upscaling. Further research would be helpful in developing the knowledge base around supporting the social and physical needs of children and young people in care, approaches to developing relationships with professionals, and promoting engagement in therapeutic work. Future research could also explore the role of gender and identify whether different types of sports maybe acceptable or more engaging to boy and girls. Future research that expands upon these findings, could explore alternative activities for young people and assess changes over time in participation.

## Data Availability

Not applicable.
